# Validity and Reliability of an On‐Bike Sensor System for the Determination of Aerodynamic Drag in Cycling

**DOI:** 10.1002/ejsc.70098

**Published:** 2025-11-28

**Authors:** James G. Hopker, Callum Barnes, Christopher R. J. Fennell, Stuart Gibson

**Affiliations:** ^1^ School of Natural Sciences University of Kent Canterbury UK; ^2^ School of Engineering, Maths and Physics University of Kent Canterbury UK

**Keywords:** coefficient drag, performance, testing, time trial, wind tunnel

## Abstract

Cycling performance is strongly influenced by aerodynamics, with most resistive drag forces being attributed to the rider. The Body Rocket device (BR) is an on‐bike sensor system that uses the same load cell technology as a wind tunnel, directly measuring real‐time aerodynamic resistance (CdA) from the rider. This study aimed to measure the validity and reliability of CdA measures from BR in two experiments. In Experiment 1, validity of BR was assessed in wind tunnel with a rod and discs of known diameter attached to the end change CdA by a known amount. Experiment 2, examined the validity and reliability of BR in a 250m indoor velodrome. Ten cyclists performed 7 identical efforts at ∼40 km/h for 10 laps of the velodrome, the first 4 to assess validity with the same rod and discs used in the wind tunnel, with the remaining 3 without changes in resistance or the riders changing position to assess reliability. Validity results demonstrated BR measured CdA to be strongly correlated with calculated CdA from the disc addition (*r* = 0.99), with the smallest identified changes being 0.002 m^2^ across wind tunnel and velodrome. Good agreement was found between theoretical and measured CdA with varying disc sizes (95% limits of agreement = −0.012 to 0.009 m^2^). A high level of reliability was demonstrated during Experiment 2 with strong intraclass correlation (0.99) and small coefficient of variation (1.67%). Findings of this study demonstrate BR is a valid and reliable device for measuring real‐time CdA during cycling in an indoor velodrome.

## Highlights

- The Body Rocket direct drag force measurement system is valid and reliable for the assessment of real‐time aerodynamic drag in cycling.
- The smallest identified change in coefficient of drag which could be detected by Body Rocket is 0.002 m^2^.
- A high level of reliability was found in the measurement of aerodynamic drag between repeated velodrome cycling efforts.

## 1 Introduction

Cycling speed is determined by a rider's power output, road surface, environmental conditions, and aerodynamic drag (CdA). The importance of aerodynamics to performance in road and track cycling is well documented with drag forces contributing to approximately 90% of resistive forces when riding at speeds above 50 km/h (Kyle and Burke 1984; Grappe et al. 1997; Martin et al. 2006). Although the bicycle provides a source of drag, the majority can be attributed to the rider, therefore it is important to reduce or minimize drag to improve performance. Indeed, previous research has demonstrated that a 16.7% reduction in drag forces can be experienced between riding on the hoods compared to a crouched position using aerobars (Barry et al. 2015). To put this in context, the reduction in drag forces is estimated to result in a reduction of 70W while maintaining a constant cycling velocity of 45 km/h.

The gold standard approach to measure aerodynamic drag is to use a force balance within a wind tunnel, which has been shown to produce highly precise and repeatable conditions (Garcia‐Lopez et al. 2008), but the high cost associated with testing makes it prohibitive for most. The wind tunnel also creates an artificial environment unlike the conditions a rider would experience if riding outside and therefore may not truly reflect race conditions which is influenced by not just aerodynamics but also factors including ambient temperature, road conditions, fatigue, rider tactics etc. Similarly other approaches for estimating CdA, such as the use of mathematical models using measured power output and speed of riders (Martin et al. 1998), dynamometric methods (Di Prampero et al. 1979), coasting‐down and deceleration methods (Kyle and Burke 1984), and digitalization or weighing photographs (Debraux et al. 2009) do not provide a direct measure of drag and, similarly to the wind tunnel, do not reflect real‐world cycling (Debraux et al. 2011).

An alternative approach is for riders to use an indoor velodrome that allows for controlled environmental conditions, a cycling power meter and, an aerodynamic sensor attached to the bicycle to provide an estimation of CdA during cycling. Indeed, various commercially available systems (such as Aerosensor or Notio Aerometer) exist for estimating CdA which consist of a pitot tube for collection of air pressure, humidity, and temperature as well as a gyroscope or accelerometer. When combined with a power meter for measuring power output and ground speed, it is then possible for these systems to indirectly compute CdA. Previous research has shown such systems to produce data which is comparable to mathematical models and capable of reliably detecting positional changes in CdA (Valenzuela et al. 2020; Kordi et al. 2022; Millour et al. 2023; Ordiñana‐Perez et al. 2023; Bruez et al. 2023). In evaluating aero devices, the majority of previous research has tended to rely on changes in rider position to determine sensitivity of detection of CdA values. Using this positional change approach, Valenzuela et al. (2020) demonstrated that the Notio Aerometer device was capable of discerning large variations in CdA (i.e., upright to aero positions with a mean change of ∼0.07 m^2^) but was not sensitive enough to identify small variations in CdA caused by helmet (∼0.008 m^2^) or wheel changes (∼0.006 m^2^). Similarly, Ordiñana‐Perez et al. (2023) suggest that the Notio Aerometer used in their study was not able to statistically discern between three different aerodynamic positions maintained by cyclists while riding within a velodrome, reporting a mean change of 0.009 m^2^. Interestingly, Ordiñana‐Perez et al. (2023) compared CdA values for Notio Aerometer to two mathematical approaches, which were also not able to statistically identify the changes in rider position. The Notio Aerometer was found to produce CdA values between 2% and 4% lower than the mathematical models. Recently, Kordi et al. (2022) aimed to compare the sensitivity of Notio Aerometer to a known CdA by using a series of discs of increasing sizes mounted to the bicycle of participants. Discs were used to add a known amount of aerodynamic drag to the system which could then be measured via the Notio Aerometer. Within a velodrome setting, Kordi et al. (2022) found the smallest change of resistance that could be identified by the Notio Aerometer was 1.2%, or 0.002 m^2^ which is much smaller than the previous studies which relied upon changes in body position. However, as outlined above, indirect drag force systems have various assumptions associated with drive train losses, rolling resistance, and weight changes relying on a technique often referred to as “Virtual Elevation” (Chung 2012). Virtual elevation necessitates the cyclist recording power output and speed over a known course. A mathematical model is then used to calculate an elevation profile based upon power, speed, mass, and air density with initial estimations for CdA and rolling resistance being adjusted iteratively until the modeled elevation profile matches the measured elevation profile, resulting in estimated values for both parameters.

Recently, an on‐bike sensor system (Body Rocket Ltd, Sussex, UK) has been developed to *directly* measure the real‐time aerodynamic drag of a rider. The system utilizes 4 force sensors that can be installed on any bicycle at the contact points between rider and bike to directly determine the forces exerted by the rider at these contact points as they move forward through the air. Therefore, using the same approach as the wind tunnel, the measurements of aerodynamic drag recorded by the system only represent those of the rider and therefore address the limitations in systems which rely on the virtual elevation method. In this respect, Body Rocket's direct force drag measurement capability avoids the need to make assumptions about rolling resistance or drivetrain losses. During dynamic cycling the varying loads are experienced on both the tires and chain due to accelerations and decelerations in bicycle speed that changes the tire losses, and a change in wheel speed, meaning assumptions of a fixed drivetrain and rolling resistance losses are incorrect, and possible sources of error. From a mathematical perspective the virtual elevation equation (Chung 2012) assumes aerodynamic drag changes with the cube of air speed whereas aerodynamic drag changes with the square of air speed (Debraux et al. 2011). Therefore, devices which rely on the virtual elevation method are susceptible to errors in their CdA calculation with even small fluctuations in speed.

However, no previous study has assessed the reliability, validity, and sensitivity of the Body Rocket system to measure CdA in static wind tunnel and free cycling conditions whereby there are dynamic yaw conditions and fluctuations in bike speed. Therefore, the aim of this study was to assess the reliability of Body Rocket during cycling as well as its validity and sensitivity to detect changes in CdA in both wind tunnel and velodrome environments.

## 2 Materials and Methods

This study comprised of two experiments to assess the validity and reliability of the Body Rocket system to measure aerodynamic drag. In the first experiment, a wind tunnel was used to determine the validity and sensitivity of the Body Rocket system. In Experiment 2, 10 male cyclists completed 8 runs of 10 laps of a 250m indoor velodrome at a constant speed to determine the validity and sensitivity as well as the reliability of the Body Rocket. The study and its methods were approved in agreement with the Declaration of Helsinki by the Research Ethics Committee of the University of Kent.

### 2.1 The Body Rocket System

**FIGURE 1:**
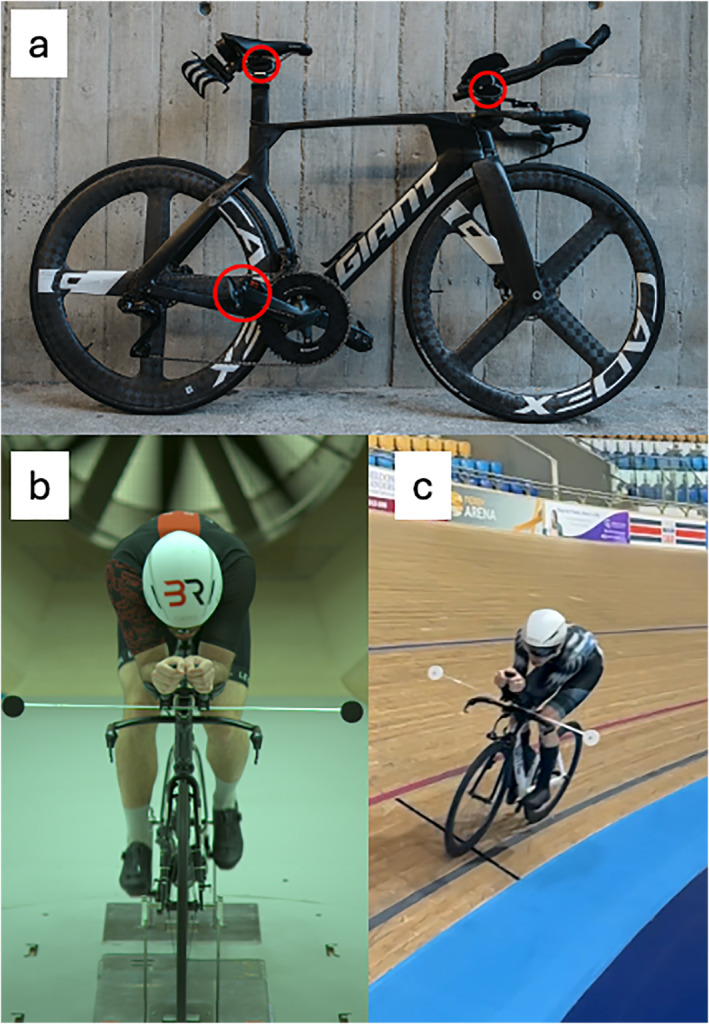
(a) A bicycle with the Body Rocket system mounted, force sensors shown circles in red located at the main points of contact for the rider on the bicycle; (b) set‐up used for wind tunnel testing with rider on bicycle; and (c) a rider performing a velodrome run with rod and discs attached.

The Body Rocket system utilizes 4 force sensors; one mounted between the basebar and aerobars, one between the saddle and seat post, and one on each pedal (see Figure 1). As each of these sensors are at the contact points of the bike, the drag of the rider can be directly determined through measuring the horizontal force at the sensor. The system also measures force moments on the saddle and handlebar. These sensors simultaneously measure forces applied in the horizontal and vertical planes, including “roll” and “pitch” moments, as well as the inclination of each sensor relative to the bike frame. Where the rider is stationary, the sum of the horizontal forces on the four sensors therefore summates to zero, with the sum of the vertical forces relative to the ground being equivalent to the weight of the rider. The sensors' accuracy is in the range of 0.05–0.1 N from calibration results (Body Rocket: unpublished data) with an acquisition frequency is 100 Hz. When the rider travels forwards through the air, the aerodynamic drag is determined by the Body Rocket system as the sensor arrangement isolates the rider from the bike at all contact points.

### 2.2 Experiment 1

To determine the validity and minimal change in CdA that can be measured by the Body Rocket system a known amount of drag was added to a bike in a wind tunnel environment (SSE, Silverstone, UK). Drag was added using a series of disc plates of increasing diameters (50, 60, 80, and 100 mm; disc Cd = 1.12) attached to the handlebars of the bicycle by a 1‐m‐long rod. The drag of each disc was calculated using a theoretical first principles approach (Fail et al. 1959) and measured via Body Rocket in the wind tunnel at a wind speed of 11.5 m/s or 41.4 km/h (equivalent to a 22 s lap time of a 250m velodrome). Drag increments between disc sizes were used to compare changes in CdA between the theorical value and that measured by Body Rocket to ascertain validity and sensitivity of the system in the wind tunnel. CdA values were obtained from 30 s recordings with a rider statically holding an aerodynamic position on the bicycle (see Figure 1b) and of the bike‐disc combination without a rider.

### 2.3 Experiment 2

Ten male cyclists volunteered to take part in this study, all having velodrome riding experience prior to participating in the study. All participants provided written informed consent after having the procedures explained to them both verbally and in writing.

The study took place in an oval 250m indoor velodrome (Derby, UK) with environmental conditions being kept as consistent as possible throughout to minimize environmental influences (e.g., one rider on a track at a time) with average environmental conditions being 20 ± 0°C, 77.9 ± 3.7% humidity, and 998 ± 0.7 kPa. Participants used a bicycle fitted with the Body Rocket instrumentation, adjusted for their body dimensions. Each cyclist completed a total of 8 runs of 10 laps holding a time trial position, one warm‐up run, three runs performed to establish the reliability with rod only and no discs, and four runs with a disc plate of 50, 60, 80, and 100 mm (see Figure 1) to assess validity. Cyclists first performed the 8 runs of the velodrome in the following order: warm‐up, reliability 1, 50 mm disc, 60 mm disc, reliability 2, 80 mm disc, 100 mm disc, and reliability 3. Drag increments between disc sizes were used to ascertain the expected changes in riders' CdA measurements compared to the Body Rocket system. During the laps, riders were asked to ride the black line at the bottom of the track and hold a set speed by matching a 22 s lap time (11.5 m/s or 41 km/h) to minimize unnecessary accelerations or decelerations, with real‐time lap splits communicated by a track‐side researcher. The first two laps of each run were used as build‐up laps to reach the target lap time with the subsequent 8 laps being used for measurement. Riders had access to real‐time speed, power, and cadence data provided by a handlebar mounted computer.

### 2.4 Statistical Analysis

Data were assessed for normality of distribution and heteroscedasticity. The relationship and 95% limits of agreement (LoA) between the Body Rocket measured change in CdA and theoretical change calculated between the various disc sizes was analyzed with Pearson's correlation coefficients, with r‐values of 0.1, 0.3, 0.5, 0.7, and 0.9 considered as small, moderate, strong, very strong, and extremely strong relationships, respectively (Hopkins et al. 2009). The 95% limits of agreement (LoA) are presented as bias ± 1.96*SD, all other data are presented as mean ± SD unless otherwise stated. The alpha level for statistical significance was set a 0.05.

Differences in speed between the different velodrome runs were assessed using repeated measures ANOVA. To assess reliability of the Body Rocket system, the within‐subject variation, expressed as a typical error, coefficient of variation (CV), and intraclass correlation coefficient, was derived from CdA data collected during the 3 repeated velodrome runs without discs attached to the rod. ICC values were interpreted as follows: low, < 0.80; moderate, 0.80–0.90; and high, > 0.90. Standardized typical errors were interpreted using modified effect size thresholds of (trivial, ≤ 0.2 and small, > 0.2–0.6); moderate, > 0.6–1.2; and large, > 1.2 (Smith and Hopkins 2011).

## 3 Results

### 3.1 Experiment 1

**FIGURE 2:**
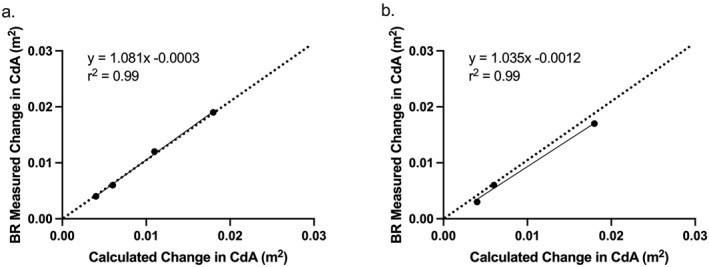
(a) Relationship between theoretical and Body Rocket measured change in CdA with increasing disc sizes; (b) Relationship between theoretical and Body Rocket measured change in CdA with rider and increasing disc sizes. 80 mm disc size omitted due to excessive rider movement during data collection. Each black dot indicates a CdA value of a disc with increasing size from 50 to 100 mm. Dotted line represents line of identity.

There was a strong near perfect correlation between theoretical changes in CdA between disc sizes and those measured by Body Rocket within the wind tunnel (*r* = 0.99 and see Figure 2). The wind tunnel comparison between the theoretical CdA calculations and those measured by the Body Rocket system demonstrated an average difference across all disc sizes of 0.002 ± 0.005 m^2^.

### 3.2 Experiment 2

**FIGURE 3:**
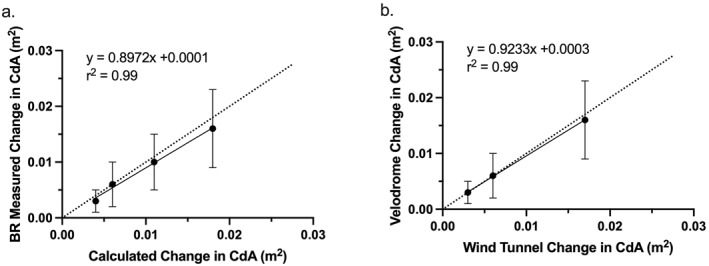
(a) Body Rocket measured versus theoretical calculated changes in CDA with increasing disc size; (b) Body Rocket measured changes in wind tunnel versus. body rocket measured changes in velodrome with increasing disc size. Data represent mean ± SD of rider cohort. Each black dot indicates a CdA value of a disc with increasing size from 50 to 100 mm. Dotted line shows line of identity.

Participants rode at a mean speed 41.4 ± 0.54 km/h (11.5 ± 0.15 m/s) during the 8 runs of the velodrome. There were no significant differences between the speeds on either the reliability runs (*p* = 0.39) or the disc test runs (*p* = 0.32). The relationship between the theoretical and Body Rocket measured changes in CdA from the different disc sizes demonstrated a large positive relationship (*r* = 0.99 and Figure 3a). The relationship between Body Rocket measured wind tunnel and velodrome changes showed a similarly large relationship (*r* = 0.99 and Figure 3b).

**FIGURE 4:**
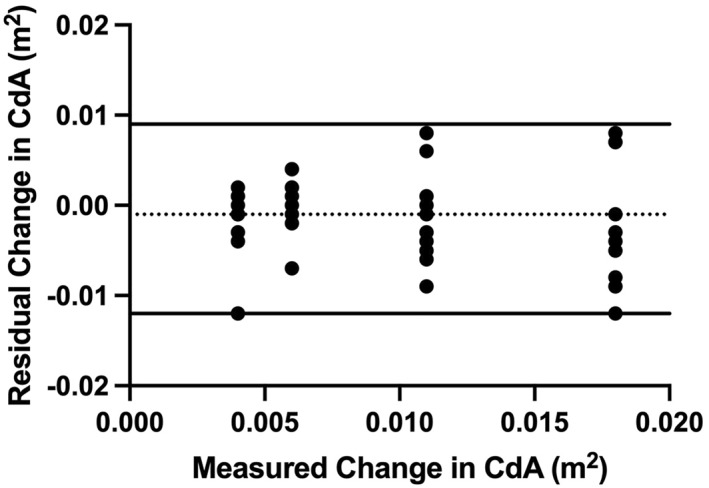
Bland–Altman plot of the difference between the theoretical and Body Rocket measured changes in CdA with increasing disc sizes during velodrome cycling. Dashed line represents the mean bias (−0.001 m^2^) and solid lines are the 95% limits of agreement.

Figure 4 shows the agreement between the theoretical and Body Rocket measured changes in CdA with increasing disc sizes, with the calculated 95% LOA of −0.012 to 0.009 m^2^.

The mean CdA measured with Body Rocket during the three reliability velodrome runs across participants was 0.208 ± 0.03 m^2^. Data were deemed to be reliable with absolute typical error being 0.004 (0.003–0.005) m^2^ and standardized typical error 0.11 (0.08–0.13), suggesting a trivial between run effect. When expressed as a CV the typical error was 1.67% (90% CI = 1.20–2.86%) and ICC = 0.99 (90% CI = 0.97–1.00).

## 4 Discussion

The current study aimed to investigate the validity, sensitivity, and reliability of a novel on bike device that uses direct drag for the real‐time measurement of a cyclist's CdA. Results of the study demonstrate that within both wind tunnel and velodrome testing conditions, Body Rocket measured CdA were strongly correlated with, and sensitive to, the calculated change arising from known quantities of drag resistance being added to the bicycle. Data suggest overall drag force sensitivity to be 0.3–0.4 N in horizontal force when comparing the smallest experimental condition changes in drag force between the rod‐only and rod + 50 mm disc tests. Body Rocket was also demonstrated to be a reliable device for the repeated measurement of CdA during indoor velodrome cycling.

The Body Rocket values in the velodrome strongly correlated with those derived from a theoretical first principles approach (*r* = 0.99) as well as from data collected in the wind tunnel (*r* = 0.99), with the regression line closely matching the line of identity (see Figure 2). In the wind tunnel, Body Rocket was able to discriminate between changes in resistive forces generated by all disc sizes with the smallest change being 0.002 m^2^ in CdA. A similarly strong correlation was found between theoretical changes in CdA with a rider in situ during wind tunnel testing. However, as shown by Figure 3, there was a tendency for Body Rocket to record lower CdA values than theoretically derived as disc size increased resulting in a deviation from the line of identity. As the discs were mounted on the rider's bicycle by a 1m long rod, turbulent airflow from the larger diameter discs could reduce resistive drag forces experienced by the rider's body as they move through the air. The resultant effect being a lower measured change in CdA from Body Rocket than the theoretical change in CdA calculated from the increased disc diameter. This effect has also been reported by Kordi et al. (2022). Another potential confounding factor in the data are the effect of the banked cornering within the Velodrome. The impact of cornering means that the speed of the two discs attached to the bicycle will be different; the outer disc being faster than the inner disc and as a result generate a greater drag force. This would result in higher drag forces being experience when cornering than on the Velodrome straights. However, as outlined in the methods, all riders were required to follow the black line at the bottom of the Velodrome to ensure the path of travel of the bicycle was the same on all runs. This means that the relative measurements to discern changes in CdA between the discs would not be affected.

In summary, the small limits of agreement and minimal bias, coupled with the strong correlation between wind tunnel and velodrome data, indicate that Body Rocket can identify changes in CdA despite small positional variations of the cyclists in the velodrome environment. As mentioned in the results unfortunately due to problems with the wind tunnel data, a direct comparison between the Wind Tunnel balance and Body Rocket was not possible. However, previous research work has suggested the Body Rocket system agrees within 2.3% with Wind Tunnel data under varied wind speed, yaw angles, and body positions (DeGolier et al. 2024).

The Bland–Altman plot shown in Figure 4 demonstrates small, but increased, residual values between wind tunnel derived and velodrome derived changes in CdA, suggestive of increased within participant variability rather than drift within the Body Rocket. Moreover, as suggested by previous research (Olds 2001), it is also likely the tendency for an underestimation of CdA may have arisen from the velodrome architecture. As the only influencing factors on Body Rocket drag readings are the air speed (and density), and the rider's position (directly affecting CdA), when the rider moves through the velodrome banking (leaning the bike) the measured air speed will be slightly faster than is experienced by their body resulting in a slightly lower value compared to the wind tunnel (Fitzgerald et al. 2019).

Comparisons of validity data across different aerodynamic devices is challenging due to different methods used to collect data. For example, Valenzuela et al. (2020) relied on changes in rider position to determine sensitivity of detection of CdA values of the Notio Aerometer, which are in turn compared to mathematical models and found the method can only identify large variations in CdA (i.e., upright to aero positions with a mean change of ∼0.07 m^2^) but was not sensitive enough to identify small variations in CdA caused by helmet (∼0.008 m^2^) or wheel changes (∼0.006 m^2^). By comparison, Kordi et al. (2022) used the same disc‐based approach in wind tunnel and velodrome environments to the current study and demonstrated a similarly high sensitivity of the Notio Aerometer device (∼0.002 m^2^). However, in contrast, Bruez et al. (2023) found a 0.058 ± 0.009 m^2^ difference between Notio Aerometer and theoretically calculated CdA values using a similar disc‐based (12 and 15 cm discs) benchmarking approach, albeit on an outdoor out‐and‐back road segment.

Results of the current study demonstrate a high level of reliability within the Body Rocket system from the repeated velodrome runs. The mean coefficient of variation from the 8‐lap effort was 1.67% (90% CI = 1.20–2.86%) with an ICC of 0.99 (90% CI = 0.97–1.00), and an absolute typical error of 0.004 m^2^ (90% CI = 0.003–0.005 m^2^). Similarly to validity comparisons, the comparison of reliability data across different aerodynamic devices is challenging due to different methods used to collect data, and differences in cycling abilities and experience of study participants. Despite all being experienced track riders, participants in the current study were all amateur cyclists competing at regional or national level. Using a similar study population, Valenzuela et al. (2020) reported an ICC of 0.92 (90%CI = 0.89–0.95) and a typical error of 0.015 m^2^ (90% CI = 0.014–0.017) from repeated velodrome runs using the Notio Aerometer. Using a higher caliber of rider (including several track cycling World Champions and medalists), Kordi et al. (2022) demonstrated a lower CV (0.47%; range = 0.16–0.69%), ICC (0.99; range = 0.98–1.00), and typical error (0.017 m^2^; range = 0.0013–0.0023 m^2^). Interestingly, when a standardized measure (Becker 1988) is used (dividing change in mean, typical error, and confidence limits by the *pure* between‐subject standard deviation for all trials), the typical error of measurement was lower in the current study than that of Kordi et al. (2022) despite the differences in study population (current study = 0.11, 90% CI = 0.08 to 0.13 vs. Kordi et al. = 0.13, and 90% CI = 0.11–0.18).

Interestingly, Bruez et al. (2023) found similar levels of reliability using the Notio Aerometer to quantify the CdA of riders in outdoor conditions (ICC = 0.98) using a flat out‐and‐back course. Therefore, despite the aforementioned lower level of sensitivity for Notio in the outdoor setting, their study demonstrated good levels of reliability, albeit with all data obtained from one participant. Evidence from Bruez et al. (2023) demonstrates some promise in the applicability of on‐bike real‐time CdA estimation devices in the field, at least from the perspective of providing repeatable data. However, a full validation study with a larger cohort of participants is required to draw firm conclusions. Similarly, data from the current study suggest that Body Rocket provides good validity, sensitivity, and reliability in wind tunnel and indoor velodrome environments. However, the application of this direct drag force measurement device is outside on the road, and so future research is required to provide such a validation within a more ecologically valid setting (e.g., with variable wind speeds and directions as well as elevation changes). In addition, the Body Rocket limits of detection for changes in CdA were not established in the current study, with 0.002 m^2^ representing the smallest change in CdA between 50 and 60 mm disc sizes. Therefore, further research is required to establish the smallest change in CdA that can be reliably detected using the Body Rocket. Future research may also examine the sensitivity of the Body Rocket system by exploring the effect of postural variations on CdA by comparing a mannequin (to exclude small postural variations) to a real rider.

## 5 Conclusions

The results of this study demonstrate that Body Rocket is a valid and reliable device for the real‐time measurement of direct drag force derived CdA during velodrome cycling. The high level of sensitivity to changes in CdA (0.002 m^2^) suggest it has the potential to provide valuable information to coaches and riders performing aerodynamic testing of changes in position or when trialling new equipment. However, further research is required to confirm its validity and reliability in outdoor conditions.

## Funding

This study was funded by a grant from Innovate UK awarded to Body Rocket Ltd and the University of Kent (Project Number: 10074539). The PhD studies of author Barnes are funded by a SEPnet SME‐DTN in partnership with Research England and Body Rocket Ltd.

## Ethics Statement

Ethical guidelines have been followed with the study design and methods being approved by the University of Kent ethics committee (Ref: 07_20_22) in agreement with the Declaration of Helsinki.

## Conflicts of Interest

The authors declare no conflicts of interest.

## Data Availability

Research data are not shared beyond that presented in the manuscript.
